# Temporal regulation of wnt signaling in alveolar development and regeneration: mechanistic insights and therapeutic advances in chronic lung diseases

**DOI:** 10.3389/fcell.2026.1914253

**Published:** 2026-08-26

**Authors:** Mingyue Ren, Guihua Song, Yan Xu, Suping Yu, Yan Zhang, Mengmeng Sun, Bingxue Zhang

**Affiliations:** 1 Department of Pediatrics, The First Affiliated Hospital of Henan University of Chinese Medicine, Zhengzhou, Henan, China; 2 School of Pediatrics, Henan University of Chinese Medicine, Zhengzhou, Henan, China

**Keywords:** bronchopulmonary dysplasia, chronic obstructive pulmonary disease, idiopathic pulmonary fibrosis, pulmonary alveoli, regenerative medicine, wnt signaling pathway

## Abstract

Wnt signaling coordinates lung specification, alveolar maturation, adult epithelial maintenance, and injury repair, but its biological output varies with developmental or disease stage, anatomical compartment, responding cell type, receptor context, and signal duration. This review organizes current evidence using a temporal-spatial framework that links Wnt ligands and receptors to source and responding cells, downstream canonical or noncanonical pathways, interacting signaling networks, and cellular outcomes. During development, mesenchymal Wnt2/2 b–β-catenin signaling specifies pulmonary endoderm, whereas canonical and noncanonical Wnt programs subsequently regulate distal progenitor expansion, branching geometry, epithelial differentiation, secondary septation, pulmonary microvascular maturation, and postnatal lung growth; maintenance of an *AXIN2* ^+^ alveolar type 2 (AT2) progenitor niche becomes a distinct function in the adult lung. After adult alveolar injury, Wnt activity is dynamically remodeled as AT2 cells proliferate and traverse damage-associated transient progenitor and keratin 8-positive (KRT8^+^) transitional states before restoring the alveolar type 1 cell layer. This framework helps reconcile apparently discordant observations in chronic lung diseases. Bronchopulmonary dysplasia disrupts Wnt timing during alveologenesis; chronic obstructive pulmonary disease can combine deficient canonical Wnt responsiveness in the alveolar compartment with Wnt5a-associated inhibition of repair and distinct airway Wnt activation; and idiopathic pulmonary fibrosis features persistent Wnt-transforming growth factor-β signaling in abnormal epithelial and fibroblast niches. Therapeutic translation should therefore avoid indiscriminate pathway activation or inhibition and instead pursue reversible, local, biomarker-guided modulation matched to disease stage, cellular compartment, receptor profile, dose, and duration.

## 1 Introduction

Wnt signaling is a conserved developmental program that coordinates embryonic patterning, tissue morphogenesis, adult stem-cell maintenance, and repair (Steinhart and Angers, 2018). Lung development progresses from foregut endoderm specification and branching morphogenesis to alveolar epithelial differentiation and postnatal alveologenesis through temporally and spatially coordinated interactions among Wnt, fibroblast growth factor, bone morphogenetic protein (BMP), Hedgehog, retinoic acid (RA), and other signaling networks (Morrisey and Hogan, 2010; Herriges and Morrisey, 2014). Disturbance of these programs contributes to bronchopulmonary dysplasia (BPD), chronic obstructive pulmonary disease (COPD), and idiopathic pulmonary fibrosis (IPF), three clinically distinct disorders that share impaired establishment, maintenance, or restoration of the alveolar gas-exchange surface (Global Initiative for Chronic Obstructive Lung Disease, 2024; Wang et al., 2025; Al Wachami et al., 2024; Raghu et al., 2022; Moreira et al., 2024).

Alveolar integrity depends on dynamic interactions among alveolar type 1 (AT1) cells, alveolar type 2 (AT2) cells, fibroblasts, endothelial cells, and immune cells (Basil et al., 2024; Song et al., 2024; Zhang and Liu, 2024). AT1 cells form the thin gas-exchange surface, whereas surfactant-producing AT2 cells act as facultative progenitors that self-renew and replenish AT1 cells after injury. Mesenchymal, vascular, and immune cells regulate these epithelial responses; failed regeneration therefore reflects the inability of a multicellular niche to coordinate a response within specific temporal windows rather than dysfunction of a single cell type.

The mammalian Wnt system comprises 19 ligands, 10 Frizzled (FZD) receptors, co-receptors including low-density lipoprotein receptor-related proteins 5/6 (LRP5/6), receptor tyrosine kinase-like orphan receptor 2 (ROR2), and receptor-like tyrosine kinase (RYK), and multiple intracellular effectors (Nusse and Clevers, 2017; Niehrs, 2012; Jin et al., 2020). Canonical Wnt signaling stabilizes β-catenin and enables T-cell factor/lymphoid enhancer-binding factor (TCF/LEF)-dependent transcription. Noncanonical pathways, including planar cell polarity (PCP) and Ca^2+^-associated branches, regulate polarity, migration, contractility, and inflammatory responses. These branches are not intrinsically beneficial or harmful; their effects depend on ligand–receptor configuration, source and responding cells, signal intensity and duration, and interactions with other pathways (Nusse and Clevers, 2017; Niehrs, 2012; Jin et al., 2020; Aros et al., 2021; Baarsma and Königshoff, 2017; Hu et al., 2021; Jirong et al., 2025).

**FIGURE 1:**
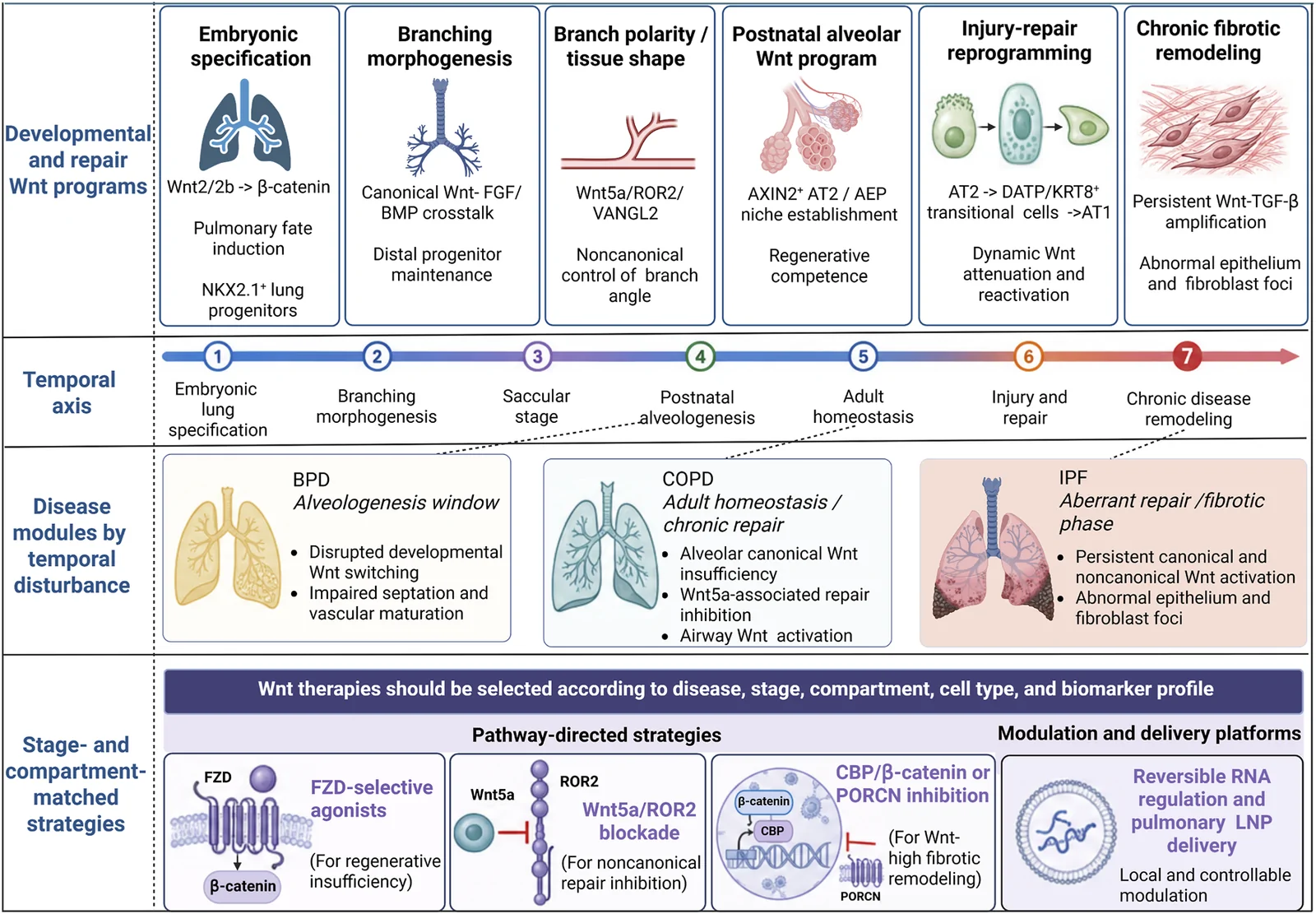
Spatiotemporal framework of Wnt signaling in lung development, regeneration, and chronic disease. Dashed lines indicate conceptual alignment across biological scales rather than established linear causal relationships.

This review therefore evaluates Wnt biology by asking five linked questions. These concern when signaling occurs and how long it persists, where it occurs, which cells produce and receive the signal through which ligand–receptor combinations, which canonical or noncanonical effectors and interacting pathways are engaged, and whether the resulting output promotes fate specification, proliferation, differentiation, morphogenesis, successful repair, or pathological remodeling. We apply this framework to developmental windows, the adult AT2 niche, injury-induced damage-associated transient progenitors (DATPs) and keratin 8-positive (KRT8^+^) transitional epithelial states, and BPD, COPD, and IPF. DATPs and KRT8^+^ cells are overlapping but non-identical intermediate states that arise during AT2-to-AT1 regeneration, whereas their persistence is associated with incomplete epithelial differentiation, maladaptive repair, and fibrosis. By connecting mechanism to disease stage and compartment, this framework clarifies why clinically meaningful Wnt intervention will require cell- and receptor-informed modulation rather than global pathway activation or inhibition. To provide an overview of the concepts discussed below, Figure 1 summarizes the spatiotemporal framework of Wnt signaling across lung development, regeneration, and chronic lung disease.

## 2 Temporal map of wnt signaling in lung development

Wnt signaling during lung development exhibits pronounced temporal and spatial specificity. Early mesenchymal-to-epithelial canonical signaling specifies pulmonary endoderm; subsequent epithelial and mesenchymal Wnt programs regulate branching and proximal-distal patterning; perinatal remodeling of Wnt activity accompanies epithelial differentiation and maturation of the gas-exchange region; and postnatal Wnt signaling contributes to alveolar epithelial expansion, secondary septation, pulmonary microvascular maturation, and lung growth, with later maintenance of a spatially restricted alveolar epithelial niche in the adult lung (Morrisey and Hogan, 2010; Herriges and Morrisey, 2014; Aros et al., 2021; He et al., 2022; Duong et al., 2022; Frank et al., 2016; Nabhan et al., 2018).

### 2.1 Combinatorial coding: ligands, receptors, co-receptors, and cell state jointly determine wnt readouts

Different Wnt ligands, FZD receptors, co-receptors, and intracellular transcriptional states do not form simple one-to-one relationships; instead, they constitute a ligand-receptor matrix that changes with developmental stage (Niehrs, 2012; Jin et al., 2020). Canonical Wnt signaling can be transmitted by ligands such as Wnt2, Wnt2b, and Wnt7b through context-dependent FZD/LRP5/6 complexes, driving β-catenin nuclear translocation. Noncanonical Wnt signaling is often associated with ligands such as Wnt5a and Wnt11 through ROR2 and VANGL planar cell polarity protein 2 (VANGL2), which regulate tissue polarity and branching direction. Multiple Wnt ligands may be co-expressed at the same stage, but differences in receptor repertoire, co-receptor availability, intracellular transcriptional state, and local matrix environment allow different cells to generate distinct readouts from the same signaling network.

Processing and transport of Wnt ligands also participate in temporal-spatial shaping. Porcupine O-acyltransferase (PORCN)-mediated lipid modification affects Wnt secretion and receptor binding; Wnt ligand secretion mediator (WLS) mediates Wnt transport from the endoplasmic reticulum/Golgi apparatus to the cell membrane; and lipoprotein particles, extracellular vesicles, and extracellular matrix can alter the diffusion radius of Wnt within tissues (Nusse and Clevers, 2017; Jin et al., 2020). Therefore, Wnt is not simply an “on-or-off” signal, but temporal-spatial information jointly constrained by secretion efficiency, diffusion range, receptor combination, and transcriptional accessibility, Thereby allowing Wnt signaling to shift from early pulmonary fate induction to compartment-specific regulation during branching morphogenesis.

### 2.2 Embryonic lung specification: Wnt2/2 b-β-catenin defines the starting point of pulmonary fate

In mice at E8.5-E9.5, splanchnic mesenchyme adjacent to the ventral foregut produces Wnt2 and Wnt2b, and the responding endoderm engages LRP-dependent canonical signaling and β-catenin-mediated transcription to induce *Nkx2.1* and pulmonary identity (Herriges and Morrisey, 2014; Aros et al., 2021; Goss et al., 2009). Goss et al. (2009) showed that combined *Wnt2/Wnt2b* deletion eliminates lung buds and tracheal structures, whereas stabilized epithelial β-catenin induces ectopic respiratory differentiation. These findings establish that mesenchymal Wnt2/Wnt2b signaling is required for lung specification and that epithelial β-catenin activation can drive respiratory differentiation when cooperating developmental signals are intact. Beyond its role in foregut epithelial specification, Wnt2-associated programs also mark multipotent cardiopulmonary progenitors that coordinate development of cardiac inflow tissues, pulmonary vasculature, and airway smooth muscle (ASM) (Peng et al., 2013).

This Wnt-dependent specification program is embedded in a broader signaling network. In RA-deficient mouse foreguts, excessive transforming growth factor-β (TGF-β) signaling suppresses local *Fgf10* expression and lung-bud induction; antibody-mediated inhibition of endogenous TGF-β restores budding, demonstrating that endogenous RA establishes a permissive signaling environment by restraining TGF-β (Chen et al., 2007). RA also represses the Wnt antagonist *Dkk1*, thereby integrating Wnt, TGF-β, and FGF10 inputs during formation of the mouse lung primordium (Chen et al., 2010). In the ventral foregut endoderm, conditional loss of the BMP type I receptors BMPR1A and BMPR1B expands SOX2, reduces NKX2-1, and causes tracheal agenesis; canonical Wnt activation cannot restore respiratory identity in the absence of these receptors, establishing a functional requirement for BMP receptor signaling alongside Wnt (Domya et al., 2011). Epithelial Hedgehog signals activate GLI-dependent programs in the adjacent foregut mesenchyme, where *Osr1* acts as a direct Hedgehog-responsive target required for normal lung-progenitor number, primary-bud branching, and pulmonary-artery differentiation (Han et al., 2017). In addition, TBX5 cooperates with Sonic Hedgehog signaling to drive mesodermal *Wnt2b* expression, providing direct evidence that Hedgehog and Wnt inputs participate in a bidirectional mesoderm–endoderm signaling loop during cardiopulmonary specification (Steimle et al., 2018). Together, these pathways delimit foregut responsiveness to canonical Wnt.

### 2.3 From pseudoglandular to canalicular stages: canonical wnt maintains distal progenitors while Wnt5a provides spatial polarity constraints

After entry into the pseudoglandular and canalicular stages, Wnt output shifts from pulmonary fate determination to compartmentalized growth and morphogenetic shaping. Mouse studies have shown that Wnt/β-catenin signaling in distal mesenchyme maintains FGF10 expression, while FGF10 promotes proliferation and migration of distal bud-tip progenitors through epithelial FGFR2b; β-catenin in epithelium and mesenchyme also regulates N-myc, BMP4, and proximal-distal transcriptional programs, maintaining distal cells in an undifferentiated and proliferative state (Shu et al., 2005; Mucenski et al., 2003). Conditional deletion of β-catenin causes disrupted proximal-distal patterning, decreased distal markers, and aberrant expansion of proximal markers, indicating that canonical Wnt is an important upstream signal for maintaining distal lung progenitor identity (Mucenski et al., 2003).

Noncanonical Wnt5a provides a complementary morphogenetic constraint. In mouse models, Li et al. found that Wnt5a deficiency causes excessive branching and morphological abnormalities of distal airways, indicating that Wnt5a constrains cell polarity, branching angle, and tissue extension through ROR2/VANGL2 and other PCP-related pathways, rather than simply inhibiting development (Li et al., 2002). Direct genetic evidence also implicates the planar-polarity component VANGL2 because *Vangl2* mutant lungs have fewer branches, perturbed epithelial cytoskeletal organization, thickened interstitial mesenchyme, and defective late saccular formation (Yates et al., 2010). Mesenchyme-specific *Foxf1* deletion reduces mesenchymal proliferation and delays branching morphogenesis; transcriptomic and chromatin-immunoprecipitation analyses identify *Wnt2*, *Wnt5a*, and *Wnt11* as FOXF1-regulated targets and *Wif1* as a directly repressed Wnt inhibitor (Ustiyan et al., 2018). These data support FOXF1-dependent regulation of mesenchymal Wnt programs. Evidence from developing mouse trachea further illustrates temporal control by extracellular Wnt modulation, as mesenchymal *Wnt5a* loss accelerates cartilaginous condensation whereas loss or inhibition of the Wnt de-acylase Notum delays condensation (Bottasso-Arias et al., 2025). Together, these findings suggest that canonical Wnt supports distal-progenitor expansion, whereas noncanonical Wnt5a imposes spatial constraints on branching.

### 2.4 Saccular stage: transition from growth programs to maturation of gas-exchange structures

The saccular stage connects airway-tree construction with formation of a thin gas-exchange surface. Across late fetal and early postnatal lung development, distal epithelial progenitors diversify into AT1 and AT2 lineages amid saccular expansion, interstitial thinning, epithelial–capillary apposition, and marked remodeling of epithelial and mesenchymal states (He et al., 2022; Duong et al., 2022). This absence of evidence for a stage-wide increase does not exclude a compartment-specific function for Wnt5a. In mice, conditional loss of mesenchymal *Wnt5a* phenocopies the impaired mesenchymal thinning and saccular expansion observed after loss of *Vangl1/2*, implicating a Wnt5a/VANGL-associated functional module in late lung morphogenesis (Paramore et al., 2024). We therefore interpret this stage as a redistribution of Wnt responsiveness among differentiating epithelial, mesenchymal, and vascular compartments rather than a binary switch from canonical to noncanonical signaling. In primary human fetal lung organoids, WNT2-expressing fibroblasts and canonical Wnt activation promote AT2 differentiation through a Wnt–NKX2.1 program, whereas NOTUM gain of function or pharmacological blockade of Wnt secretion reduces SFTPC and other AT2-lineage markers (Lim et al., 2023); complementary mouse experiments show that Wnt5a gain of function represses AT2-lineage gene expression in a cell-state-dependent manner while promoting AT1 programs (Li et al., 2022). Together, these findings show that the level and branch of Wnt signaling influence AT2 differentiation, expansion, and AT1/AT2 fate balance in a developmental-window- and cell-state-dependent manner.

Species differences are particularly important at this stage. In mice, saccular maturation occurs late in gestation and alveologenesis is predominantly postnatal, whereas human alveolar formation begins before birth and continues through infancy and early childhood (Duong et al., 2022; Thébaud et al., 2019; Ambalavanan et al., 2026). Because mouse E17.5–P5 and P5–P30 windows do not map directly onto preterm human lung development, findings from mouse developmental and neonatal hyperoxia models require validation in preterm infant lung tissue, noninvasive biomarkers, and human-derived lung organoids before informing BPD therapeutic-window design.

### 2.5 Postnatal wnt signaling during alveologenesis: epithelial expansion, secondary septation, pulmonary microvascular maturation, and lung growth

Postnatal alveologenesis depends on coordinated expansion of the alveolar epithelium and organization of new secondary septa. In *Axin2* reporter mice, canonical Wnt-responsive cells become enriched in the SFTPC^+^ AT2 lineage; β-catenin activation promotes AT2 proliferation and clonal expansion, whereas its deletion reduces AT2 expansion and biases descendants toward an AT1 fate (Frank et al., 2016). In parallel, mesenchymal WNT5A activates ROR2–VANGL2/PCP signaling in alveolar epithelial cells and fibroblasts/myofibroblasts. Downstream actomyosin remodeling links epithelial shape and PDGFA trafficking to the differentiation and migration of PDGFRα^+^/ACTA2^+^ myofibroblasts toward nascent septal tips (Zhang et al., 2020; Li et al., 2020). Together, these canonical and noncanonical programs coordinate AT2 expansion with secondary septation.

Concurrently, developing septa mature into functional gas-exchange units through stromal and microvascular coordination. Conditional WLS deletion in NKX2-1^+^/SFTPC^+^ or AGER^+^ epithelium produces enlarged saccules and impaired mesenchymal or endothelial maturation, establishing epithelial Wnt secretion as a cross-compartmental developmental input (Fang et al., 2022). In pulmonary microvascular endothelial cells, LRP5-β-catenin-TIE2 signaling promotes capillary-network formation and alveolar septation, with the angiopoietin-1/angiopoietin-2 balance tuning its output (Mammoto et al., 2012). Together, these compartment-specific Wnt programs coordinate epithelial, septal, and microvascular maturation.

These stage-specific developmental Wnt modules are summarized in Table 1.

**TABLE 1:** Stage-resolved Wnt signaling modules during lung development and postnatal alveologenesis

Developmental stage	Cellular, tissue, and environmental context	Wnt ligands, receptors, and signaling branch	Major biological functions	Key interacting pathways	Ref.
Respiratory specification and cardiopulmonary co-development	WNT2/2 B-producing splanchnic and cardiopulmonary mesenchyme signal to ventral foregut endoderm and mesodermal derivatives at mouse E8.5–E9.5	WNT2/2B; LRP5/6; canonical β-catenin signaling	*Nkx2.1* induction, lung/tracheal specification, and coordination of pulmonary vasculature, ASM, and cardiac inflow tissues	RA–TGF-β–*Dkk1*/FGF10; BMPR1A/B–SOX2; Hedgehog–GLI–OSR1; TBX5–WNT2/2 B	(Goss et al., 2009; Peng et al., 2013; Chen et al., 2007; Chen et al., 2010; Domya et al., 2011; Han et al., 2017; Steimle et al., 2018)
Pseudoglandular–canalicular branching	Bidirectional signaling in the distal bud-tip epithelial–mesenchymal niche; extracellular Wnt availability is locally modulated by NOTUM	Canonical β-catenin signaling; WNT5A-associated noncanonical signaling; VANGL2/PCP	Distal progenitor maintenance, proximal–distal patterning, and control of branching growth and polarity	FGF10–FGFR2b, N-myc, BMP4, FOXF1/WIF1, and NOTUM	(Shu et al., 2005; Mucenski et al., 2003; Li et al., 2002; Yates et al., 2010; Ustiyan et al., 2018; Bottasso-Arias et al., 2025)
Saccular maturation	Differentiating distal epithelium, interstitial mesenchyme, and microvasculature during saccular expansion, interstitial thinning, and epithelial–capillary apposition	Mesenchymal WNT5A/VANGL-associated noncanonical signaling; canonical Wnt–NKX2.1 signaling modulated by NOTUM	Sacculation, interstitial thinning, AT1/AT2 differentiation, and gas-exchange maturation	NKX2.1/NOTUM, VANGL-dependent PCP, matrix remodeling, and vascular apposition	(He et al., 2022; Duong et al., 2022; Paramore et al., 2024; Lim et al., 2023; Li et al., 2022)
Postnatal alveologenesis: AT2 expansion and fate balance	Canonical Wnt-responsive *AXIN2* ^+^/SFTPC^+^ AT2 subsets during late sacculation and postnatal lung growth	Canonical β-catenin–TCF/LEF signaling	AT2 proliferation and clonal expansion, AT1/AT2 fate balance, and epithelial contribution to lung growth	FGF18, Hedgehog, BMP4, TGF-β2, and VEGF-associated programs	(Frank et al., 2016)
Postnatal alveologenesis: secondary septation	Mesenchymal WNT5A from alveolar fibroblasts/myofibroblasts acts on alveolar epithelium and PDGFRα^+^/ACTA2^+^ cells at nascent septal tips	WNT5A–ROR1/2–VANGL2/PCP signaling	Epithelial shape change, myofibroblast differentiation and migration, and secondary-septum initiation and elongation	PCP-dependent actomyosin remodeling and WNT5A–PDGFA/PDGFRA feedback	(Zhang et al., 2020; Li et al., 2020)
Postnatal alveologenesis: epithelial–vascular coordination	NKX2-1^+^/SFTPC^+^ and AGER^+^ epithelium signal to mesenchyme and endothelium; LRP5-expressing endothelial cells support the neonatal capillary niche	WLS-dependent epithelial Wnt secretion; endothelial LRP5–β-catenin–TIE2 signaling	Stromal and endothelial maturation, capillary-network formation, and coordinated alveolar septation	Ang1/Ang2–TIE2, mesenchymal α-SMA/collagen programs, and endothelial-to-mesenchymal-transition-like remodeling	(Fang et al., 2022; Mammoto et al., 2012)

Collectively, these studies establish stage- and compartment-specific Wnt functions across lung development and provide a foundation for spatially resolving endogenous receptor usage in the human lung.

## 3 Wnt signaling in adult alveolar stem-cell niches

In the adult alveolus, Wnt signaling shifts from controlling morphogenesis to coordinating injury repair through stage- and context-dependent epithelial-stromal-immune interactions.

### 3.1 *AXIN2* ^+^ AT2 stem-cell niche: from cell type to spatial ecosystem

The adult lung turns over slowly at homeostasis but retains regenerative capacity through AT2-cell self-renewal and differentiation into AT1 cells, thereby restoring the gas-exchange barrier (Barkauskas et al., 2013; Desai et al., 2014). However, AT2 cells are not a homogeneous population. Mouse single-cell transcriptomic, lineage-tracing, and organoid studies have shown that *AXIN2* ^+^ AT2 cells have higher clone-forming capacity and stronger contribution after injury (Nabhan et al., 2018; Zacharias et al., 2018). Human lung cell atlases indicate that the AT2 cell-surface phenotype defined by HTII-280 immunoreactivity, the expression levels of surfactant protein C (SFTPC) and transmembrane 4 L six family member 1 (TM4SF1), lipid metabolic state, mitochondrial function, and aging-related features further subdivide AT2 heterogeneity (Travaglini et al., 2020; Kadur et al., 2022). Therefore, direct extrapolation of the mouse *AXIN2* ^+^ alveolar epithelial progenitor (AEP) concept to human COPD, IPF, or preterm-infant BPD therapy requires validation using spatial omics and functional organoid models based on human samples.

The function of *AXIN2* ^+^ AT2 cells depends on their niche. PDGFRα^+^ fibroblasts provide paracrine Wnt signals, while lipid-associated fibroblasts, myofibroblast-like cells, endothelial cells, and immune cells jointly shape local signaling intensity (Nabhan et al., 2018; Zepp et al., 2017). The spatial distance between AT2 cells and fibroblasts is a key parameter determining the intensity and duration of Wnt signaling. Excessive distance may cause Wnt insufficiency, whereas aberrantly activated fibroblasts may couple Wnt with TGF-β, matrix stiffening, and inflammatory signals, thereby redirecting repair toward pathological outcomes.

### 3.2 The R-spondin/ZNRF3/RNF43 axis: a critical regulator of wnt sensitivity

The same concentration of Wnt ligands can generate different outputs across cell types and disease states, partly because of differences in receptor stability and cellular sensitivity. The R-spondin/ZNRF3/RNF43 axis provides receptor-level control for the Wnt pathway. ZNRF3 and RNF43 are membrane E3 ubiquitin ligases that promote endocytosis and degradation of FZD receptors; after R-spondin binds to LGR receptors, it inhibits ZNRF3/RNF43 activity, allowing FZD receptors to remain on the cell surface and amplifying Wnt responses (de Lau et al., 2014). Alveolar organoid culture often requires exogenous Wnt or R-spondin support, functionally indicating that maintenance of AT2 stemness depends not only on the presence of Wnt ligands, but also on receptor stability, intracellular transcriptional state, and local extracellular matrix.

Dysregulation of this axis in either direction may be detrimental. Increased ZNRF3/RNF43 activity may impair AT2 responses to niche-derived Wnt, whereas excessive R-spondin or FZD-stabilizing signals may prolong β-catenin output and increase the risk of abnormal proliferation or fibrosis. At present, the cellular sources and post-injury dynamics of R-spondin in adult alveolar niches, particularly in COPD and IPF, remain incompletely defined.

### 3.3 Injury-induced transitional alveolar epithelial states and dynamic wnt remodeling

Single-cell transcriptomic, lineage-tracing, and organoid studies show that alveolar epithelial regeneration after injury can involve a spectrum of overlapping transitional states, collectively exhibiting partially shared expression of KRT8, keratin 18 (KRT18), and claudin-4 (CLDN4), together with enrichment of p53-and HIF-related signaling programs and inflammatory-response programs (Choi et al., 2020; Strunz et al., 2020; Kobayashi et al., 2020). As summarized in Supplementary Table S1, DATPs, the Krt8^+^ alveolar differentiation intermediate (ADI), and the pre-alveolar type-1 transitional cell state (PATS) were defined using partly distinct experimental frameworks and criteria and should therefore be regarded as overlapping but non-equivalent transitional epithelial states, a distinction that is important when interpreting their relationship with Wnt activity.

As reviewed by Raslan and Yoon (2020), Wnt activity is dynamically remodeled during alveolar regeneration, and the direction and magnitude of these changes vary with the injury model, repair stage, cell type, and niche context. Accordingly, phenotypically similar transitional epithelial states may be associated with different patterns of Wnt activity.

### 3.4 Broader niche regulation: autocrine wnt, immune licensing, and metabolic inputs

The adult alveolar Wnt niche is not limited to a binary epithelial-fibroblast relationship, but is shaped jointly by epithelial, mesenchymal, immune, endothelial, and metabolic signals. Injury-induced mesenchymal-epithelial niches can coordinate local regenerative responses (Jones et al., 2024). In addition to paracrine Wnt from fibroblasts, AT2 cells can also participate in Wnt signal generation. Chen et al. reported that activation of the α7 nicotinic acetylcholine receptor (α7nAChR) enhances an autocrine Wnt7b loop in AT2 cells and promotes alveolar regeneration, indicating that AT2 cells in the injured environment are not passive recipients of niche signals but can transiently form self-reinforcing regenerative modules (Chen et al., 2023). Immune cells also influence Wnt-dependent repair. Ruscitti et al. found that recruited atypical Ly6G^+^ macrophages license alveolar regeneration, and Wei et al. further suggested that macrophage peroxisome-mediated lipid metabolism participates in alveolar repair (Ruscitti et al., 2024; Wei et al., 2025). Together, these studies indicate that Wnt output depends on inflammatory intensity, matrix stiffness, oxygen tension, and cellular metabolic state (Wang et al., 2024).

## 4 Dysregulation of the wnt pathway in chronic lung diseases

BPD, COPD, and IPF represent three distinct temporal patterns of Wnt temporal disturbance: developmental interruption, failure of adult homeostatic maintenance, and aberrant post-injury repair. These alterations are not uniformly pro-regenerative or pro-fibrotic but depend on model, species, time point, spatial compartment, and cellular state.

### 4.1 BPD: disruption of wnt remodeling during the alveologenesis window

BPD mainly occurs from late gestation through the postnatal alveologenesis window, and its core pathology is arrested alveologenesis and impaired microvascular development (Thébaud et al., 2019; Ambalavanan et al., 2026). Preterm birth exposes the developing lung to extrauterine hyperoxia, mechanical ventilation, infection, inflammation, and nutritional fluctuations. These factors can disrupt postnatal Wnt-dependent epithelial, mesenchymal, and vascular programs, leading to deficient secondary septation, reduced alveolar number, enlarged airspaces, simplified capillary networks, and subsequent impairment of alveolar niche maturation. In studies of human preterm infants and developmental lung injury models, Sucre et al. suggested that upregulation of mesenchymal Wnt5a mediates hyperoxia-induced injury in the developing lung, showing that premature dominance of noncanonical Wnt can disrupt alveologenesis (Sucre et al., 2020). This observation reinforces the importance of appropriately timed noncanonical Wnt signaling for proper tissue patterning.

However, Wnt abnormalities in BPD should not be simplified as either “canonical Wnt insufficiency” or “excessive β-catenin activation.” In experimental BPD models, Alapati et al. found that inhibition of β-catenin signaling improves alveolarization and reduces pulmonary hypertension (Alapati et al., 2014). Interactions among peroxisome proliferator-activated receptor-γ (PPARγ), TGF-β, and Wnt may also determine the shift of lipofibroblasts toward a myofibroblast-like state, thereby influencing alveolar septation (Lecarpentier et al., 2019; Dasgupta et al., 2009). These results indicate that the direction of Wnt effects in BPD depends on the intensity and duration of hyperoxic exposure, inflammatory background, and cell type.

By contrast, AT2-specific β-catenin deletion during physiological postnatal alveologenesis reduced AT2 proliferation and expansion (Frank et al., 2016). Canonical Wnt activation also partially restored epithelial niche support by hyperoxia-exposed PDGFRα^+^ fibroblasts in organoid assays (Riccetti et al., 2022). Together, these findings suggest that β-catenin inhibition may benefit early hyperoxic stress, whereas excessive Wnt suppression may impair AT2-dependent recovery. Because the timing of alveologenesis differs between mice and humans, these findings require validation in human lung tissues and organoid models.

### 4.2 COPD: coexistence of canonical wnt insufficiency in the alveolar compartment and wnt activation in the airway compartment

In COPD, Wnt dysregulation affects adult alveolar maintenance and chronic repair (Qu et al., 2019). Long-term cigarette smoke exposure, oxidative stress, chronic inflammation, and cellular senescence jointly weaken canonical Wnt signaling in the alveolar compartment; cigarette smoke can also restrict the ability of mesenchymal cells to support lung epithelial organoid formation (Khedoe et al., 2023). In human COPD lung tissues and experimental models, Skronska-Wasek et al. found that reduced *FZD4* expression is associated with weakened canonical Wnt/β-catenin activity and that cigarette smoke can reduce *FZD4* expression through epigenetic mechanisms such as promoter methylation (Skronska-Wasek et al., 2017). Given that the FZD4 receptor participates in alveolar epithelial responses to niche-derived Wnt, its downregulation may weaken AT2 self-renewal and AT1 replenishment.

At the same time, Wnt5a is often increased in COPD lung tissue. Baarsma et al. proposed that mesenchyme-derived Wnt5a/5 b can impair the repair of alveolar epithelial progenitors, potentially by antagonism of β-catenin stabilization via ROR2-related pathways, enhancement of inflammation and matrix remodeling, and alteration of epithelial-mesenchymal signaling direction (Baarsma et al., 2017). Animal emphysema studies have shown that activation of canonical Wnt/β-catenin can attenuate experimental emphysema-like destruction, while inhibition of lymphotoxin β receptor (LTβR) signaling can unleash Wnt-induced regenerative potential in the lung (Kneidinger et al., 2011; Conlon et al., 2020). Together, these findings support the concept that canonical Wnt insufficiency in the COPD alveolar compartment contributes to repair failure.

By contrast, Carlier et al. observed activation of the canonical Wnt pathway in human COPD airway epithelium (Carlier et al., 2020). In addition, studies of COPD airway remodeling suggest that Wnt may participate in basal-cell proliferation, mucous metaplasia, and small-airway lesions (Liu et al., 2023). Whole-lung homogenates or single-compartment analyses are insufficient to determine the direction of Wnt therapy. A more reasonable interpretation is “compartment-specific Wnt dysregulation”: canonical Wnt insufficiency in the alveolar compartment drives AT2 regeneration failure and emphysema-like destruction, whereas Wnt activation in the airway compartment may reflect compensatory proliferation or pathological remodeling under chronic stimulation.

### 4.3 IPF: persistent activation of developmental wnt repair programs in adult fibrotic foci

In IPF, abnormal epithelium, Krt8+ transitional states, and fibroblast foci jointly form Wnt-TGF-β-related signaling axes (Zhou et al., 2024). Unlike canonical Wnt insufficiency in the COPD alveolar compartment, IPF lung tissue—especially fibroblast foci and hyperplastic abnormal epithelial regions—exhibits nuclear β-catenin accumulation documented by Chilosi et al., upregulation of multiple Wnt ligands/receptors, and aberrant expression of Wnt target genes (Chilosi et al., 2003; Königshoff et al., 2009; Königshoff et al., 2008). Königshoff et al. further demonstrated that WNT1-inducible signaling pathway protein 1 (WISP1), also termed cellular communication network factor 4 (CCN4), is an important downstream mediator of the pro-fibrotic effects of Wnt/β-catenin, promoting epithelial-mesenchymal transition-like changes, fibroblast proliferation, and extracellular matrix deposition (Königshoff et al., 2009).

Wnt and transforming growth factor-β (TGF-β) are interconnected through several cell- and state-specific axes in IPF. TGF-β promotes fibroblast activation and extracellular matrix deposition by altering Wnt ligands, extracellular modulators, receptors, and downstream transcriptional programs; Wnt/β-catenin or noncanonical outputs can in turn reinforce profibrotic cell states. LRP5-dependent signaling contributes causally to fibrosis in mouse models and is associated with disease progression in human IPF (Lam et al., 2014). Extracellular-vesicle WNT5A (EV-WNT5A) extends noncanonical communication between fibrotic cells and can regulate TGF-β-induced matrix production (Martin-Medina et al., 2018; Kumawat et al., 2013). FZD2 integrates canonical and noncanonical programs in experimental lung fibrosis, while a fibroblast-dependent TGF-β1/sFRP2 axis promotes epithelial metaplasia in human interstitial lung disease samples, AT2-fibroblast organoids, and precision-cut lung slices (Zhou et al., 2025; Cohen et al., 2024).

Secreted Frizzled-related protein 1 (SFRP1) is classically recognized as an extracellular Wnt antagonist, but its function in the fibrotic lung is cell-state- and context-dependent. Mayr et al. (2024) identified an early Sfrp1^+^ transitional fibroblast state in regenerating mouse lungs that preceded the emergence of collagen triple helix repeat containing 1-positive (Cthrc1^+^) myofibroblasts. In the same study, SFRP1^+^ fibroblasts were enriched in mildly affected regions of human IPF lungs, whereas end-stage regions contained more ACTA2^+^ myofibroblasts localized to fibroblast foci. Mechanistically, TGF-β1 downregulated SFRP1 in primary human lung fibroblasts during acquisition of an invasive CTHRC1^+^ phenotype. SFRP1 depletion increased basal invasion, reconstitution partially rescued the knockdown phenotype, and exogenous SFRP1 treatment reduced invasion, with these effects partly involving regulation of RHOA pathway activity (Mayr et al., 2024). Collectively, these findings identify SFRP1 as a state-specific regulator of fibroblast invasiveness during the transition to pathogenic myofibroblasts and suggest a potential strategy for limiting fibroblast-foci formation, rather than supporting broad systemic Wnt inhibition in IPF.

Preclinical evidence indicates that selective inhibition of pathological Wnt output can attenuate fibrosis, as exemplified by reversal of experimental pulmonary fibrosis after CREB-binding protein (CBP)/β-catenin inhibition (Henderson et al., 2010). However, alveolar epithelial repair requires stage-specific Wnt reactivation. Wnt5a is likewise context-dependent: in COPD, it can suppress alveolar repair; in IPF, EV-WNT5A may spread fibrotic signaling; during normal development, it is necessary for branching polarity and tissue-shape constraints. Thus, therapeutic targets in IPF should be specific ligand-receptor-cell combinations active during the fibrotic stage rather than “all Wnt.”

### 4.4 Disease stage, wnt disturbance, and pathological consequences

BPD, COPD, and IPF can be placed within a shared temporal Wnt coordinate system, but their major directions of disturbance, affected compartments, and pathological consequences differ. Supplementary Table S2 applies a common framework to each disease: temporal stage, producing and responding compartments, representative ligands and receptors, downstream branch, crosstalk, biological consequence, and evidence boundary.

The comparison shows why whole-lung measurements cannot determine a therapeutic direction. BPD combines developmental vulnerability with model- and cell-dependent β-catenin effects; COPD separates alveolar regenerative insufficiency from airway activation; and IPF concentrates persistent Wnt output in abnormal epithelial and fibroblast niches. These differences motivate the treatment scenarios considered below.

## 5 Therapeutic strategies: from tool classification to disease-stage-compartment matching

Wnt therapeutic strategies should move from conventional drug-disease matching toward drug-cell state-temporal window-compartment matching. For each pathological condition, a translational strategy must define the therapeutic objective, molecular and cellular target, evidence level, delivery route, key risks, and candidate biomarkers for target or patient selection.

### 5.1 Matching wnt disturbance patterns with therapeutic strategies

Therapeutic selection should begin with the dominant disturbance identified in Supplementary Table S2 and then match the intervention to its evidence level and safety constraints. Table 2 compares therapeutic goals, candidate strategies, risks, and target-selection biomarkers; most Wnt-directed approaches remain preclinical. Future studies should incorporate prospective patient stratification and pharmacodynamic monitoring while clearly distinguishing validated from exploratory biomarkers.

**TABLE 2:** Wnt disturbance patterns, therapeutic strategies, and target-selection biomarkers.

Therapeutic scenario	Major therapeutic goal	Potential strategies and evidence level	Key risks	Candidate target-selection or pharmacodynamic biomarkers
BPD alveologenesis window	Restore developmental signaling balance while reducing hyperoxic and inflammatory injury	Established supportive care includes optimized oxygen and respiratory management; vitamin A is clinically used in selected preterm infants, whereas direct FZD-selective modulation remains hypothetical	Excessive β-catenin activation could disturb epithelial differentiation or vascular development; excessive inhibition could impair alveologenesis	Developmental age, oxygen exposure, imaging indices of alveolar and vascular growth; tissue or experimental *AXIN2* and Wnt5a measurements remain research biomarkers
COPD with alveolar regenerative insufficiency	Transiently enhance canonical Wnt responsiveness in AT2 cells and mitigate Wnt5a-associated repair inhibition	Pharmacological canonical Wnt/β-catenin activation has elicited repair-associated responses in COPD patient-derived three-dimensional lung tissue cultures. Biomarker-matched strategies could build on this evidence through FZD5- or FZD6-selective agonism or epithelium-targeted CasRx-mediated canonical Wnt signaling modulation for an alveolar epithelial canonical-Wnt-low phenotype, together with proposed short-course WNT5A–ROR2 pathway blockade for a noncanonical-Wnt-high phenotype. These precision approaches are supported by mechanistic and preclinical proof of concept and provide candidates for COPD-specific validation	Airway Wnt activation, off-target stromal or endothelial responses, persistent proliferation, and oncogenic risk	Exploratory markers include reduced alveolar epithelial *FZD4* expression and β-catenin activity, preserved *FZD5/FZD6* expression and SFTPC/HTII-280-defined AT2 reserve, KRT8/CLDN4 transitional-state and senescence burden, and epithelial ROR2 together with compartment-resolved WNT5A. *AXIN2* may support pharmacodynamic monitoring of FZD agonism but may be difficult to interpret when directly targeted by CasRx. These candidate biomarkers may support future patient stratification and pharmacodynamic monitoring but require prospective validation in COPD
Active fibrotic IPF	Suppress pathological Wnt output while preserving residual epithelial repair	CBP/β-catenin and PORCN inhibitors are preclinical; FZD2, LRP5, WNT5A, and SFRP-linked strategies are exploratory; nintedanib and pirfenidone are established antifibrotics but not Wnt-targeted drugs	Impaired AT2 repair, systemic intestinal and skeletal toxicity, drug interactions, and selection of resistant or alternative profibrotic states	Nuclear β-catenin, WISP1/CCN4, extracellular-vesicle WNT5A, SFRP1/SFRP2 states, TGF-β activity, CTHRC1^+^ fibroblasts, and fibroblast-foci burden require prospective validation
Post-injury recovery	Support epithelial regeneration without locking cells in proliferative or transitional states	Reversible RNA regulation, receptor-selective agonists, and organoid-defined combinations remain preclinical	Persistent Krt8^+^/CLDN4^+^ states, fibrotic niche conversion, and abnormal proliferation	Spatial Wnt-response markers, Krt8/CLDN4 transitional-state burden, AT1 maturation markers, and lineage-specific proliferation indices

### 5.2 Restoring developmental timing rather than unidirectional wnt activation during the BPD alveologenesis window

During the BPD alveologenesis window, the key issue is not simply whether β-catenin should be enhanced, but whether the developmental transition between canonical and noncanonical Wnt programs has been distorted by preterm-birth-related hyperoxia, mechanical ventilation, and inflammation. Current clinical treatment of BPD remains based on prevention of preterm birth, optimization of respiratory support, reduction of oxygen toxicity, nutritional support, and inflammation control (Thébaud et al., 2019; Ambalavanan et al., 2026). Vitamin A/RA-related strategies may indirectly influence alveologenesis by promoting epithelial-mesenchymal maturation and alveolar septation, but their precise relationship with Wnt timing still requires validation in clinical samples and human-derived models.

The benefit of β-catenin inhibition in hyperoxic animal models argues against directly importing the Wnt-enhancement logic proposed for COPD (Alapati et al., 2014). A stage-stratified BPD strategy remains a hypothesis: acute hyperoxia/inflammation may favor injury control and reduction of aberrant Wnt5a output, whereas recovery-phase Wnt support should be tested only where AT2-niche canonical insufficiency is demonstrated. Neither sequence has been validated in preterm infants.

### 5.3 COPD alveolar regeneration insufficiency: AT2-biased, short-course, and reversible canonical wnt enhancement

The therapeutic objective in COPD with alveolar regenerative insufficiency would be to restore AT2-cell responsiveness to niche-derived Wnt and mitigate Wnt5a-associated inhibition of repair without activating Wnt throughout the lung. Direct evidence from COPD patient-derived three-dimensional lung tissue cultures supports the regenerative premise of this strategy. Uhl et al. showed that pharmacological canonical Wnt/β-catenin activation with the glycogen synthase kinase-3β inhibitors lithium chloride and CHIR99021 increased alveolar epithelial marker expression, decreased matrix metalloproteinase-12 expression, and attenuated pathological tissue features (Uhl et al., 2015). Frizzled-selective agonists extend this regenerative principle toward receptor-selective, cell-informed regulation. In mice subjected to repeated bleomycin lung injury, FZD-selective agonists developed by Nabhan et al. showed that both Fzd5ag and Fzd6ag activated canonical Wnt signaling in alveolar epithelial stem cells, increased their activity, and improved survival, whereas only Fzd6ag promoted an alveolar fate in airway-derived progenitors (Nabhan et al., 2023).

Complementing receptor-selective agonism, Shen et al. showed that intratracheal AAV6-CasRx-mediated depletion of *Axin1* and *Axin2* transcripts reversibly activated canonical Wnt/β-catenin signaling in the mouse lung epithelium, promoted AT2-cell proliferation and alveolar regeneration, and attenuated fibrosis in both preventive and therapeutic protocols in a bleomycin-induced lung injury model (Shen et al., 2024). Collectively, the human COPD tissue, FZD-agonist, and CasRx studies support a translational framework that combines restoration of canonical Wnt responsiveness with receptor or epithelial targeting and reversible pathway modulation. CasRx translation will additionally require evaluation of redosing feasibility and vector immunity.

Alongside canonical Wnt enhancement, Wnt5a/ROR2 blockade may provide a complementary therapeutic route for molecularly selected COPD subgroups, with the goal of mitigating noncanonical Wnt-mediated suppression of alveolar repair rather than directly activating β-catenin. Because Wnt5a still has important physiological functions in normal tissue polarity and injury-induced tissue shaping, long-term global inhibition is inappropriate. An ideal strategy may involve short-course, alveolar-biased Wnt5a blockade combined with anti-inflammatory, antioxidant, and smoking-cessation interventions, but only in molecularly defined subgroups with evidence of alveolar rather than airway-dominant Wnt disturbance.

### 5.4 Active fibrotic phase of IPF: inhibiting pathological wnt-tgf-β reinforcing axes while preserving epithelial repair windows

The therapeutic goal in active fibrotic IPF is to attenuate pathological Wnt output in fibroblast foci and abnormal epithelium while preserving stage-specific signaling required for residual AT2 repair. PRI-724 is a small-molecule antagonist of the interaction between β-catenin and CREB-binding protein (CBP), designed to alter canonical Wnt transcriptional output rather than block β-catenin globally (Okazaki et al., 2019). Henderson et al. established antifibrotic activity for CBP/β-catenin inhibition in experimental pulmonary fibrosis (Henderson et al., 2010). Okazaki et al. subsequently showed that delayed PRI-724 treatment ameliorated bleomycin-induced fibrosis in mice, with changes in alveolar macrophage populations and TGF-β1 expression (Okazaki et al., 2019). These are preclinical findings; PRI-724 is not an approved treatment for IPF, and its cell selectivity, optimal treatment window, and long-term safety in chronic human disease remain undefined.

PORCN inhibitors, such as LGK974 and, ETC-159, block lipid modification and secretion of Wnt ligands, thereby reducing multiple Wnt signals at the source and theoretically applying to Wnt-high fibrotic subgroups (Liu et al., 2013). Their risk lies in the inability to distinguish harmful fibrotic Wnt from necessary homeostatic/regenerative Wnt; long-term use may impair repair functions in the intestine, bone, hematopoietic system, and alveolar epithelium. Therefore, any use in IPF should be short-course, local, or biomarker-matched rather than broad and prolonged.

FZD2, LRP5, extracellular-vesicle WNT5A, and SFRP-regulated states offer more selective experimental targets. These approaches should be distinguished from the approved antifibrotic agents nintedanib and pirfenidone. Nintedanib is an oral small-molecule intracellular tyrosine kinase inhibitor used to slow disease progression in IPF (Wollin et al., 2015). It inhibits platelet-derived growth factor receptors, fibroblast growth factor receptors, and vascular endothelial growth factor receptors, thereby limiting fibroblast proliferation, migration, differentiation, and extracellular-matrix deposition (Wollin et al., 2015). Pirfenidone is an oral synthetic pyridone antifibrotic approved for IPF; it has pleiotropic effects on TGF-β-associated fibroblast activation, fibroblast-to-myofibroblast transition, and collagen and other extracellular-matrix production, but no single definitive molecular target has been established (Ruwanpura et al., 2020). Phase III trials showed that both drugs slow forced vital capacity decline or disease progression in IPF, but neither is curative (Richeldi et al., 2014; King et al., 2014). If Wnt-modulating agents are combined with nintedanib or pirfenidone in future studies, Wnt–TGF-β activity, fibroblast-foci burden, epithelial transitional-state proportion, and lung-function decline should be treated as exploratory monitoring endpoints rather than inferred from current clinical guideline recommendations.

### 5.5 Post-injury recovery phase and delivery platforms: reversible, local, and monitorable

Evidence from experimental pulmonary fibrosis and alveolar regeneration supports a stage-dependent therapeutic framework of “initial decline, resetting, and subsequent moderate activation,” in which excessive profibrotic Wnt output is first attenuated, the evolving injury niche is then reassessed, and regenerative canonical Wnt signaling is moderately restored when epithelial repair remains insufficient (Henderson et al., 2010; Shen et al., 2024). In a mouse diphtheria-toxin alveolar epithelial-ablation model, AT2-cell Wnt7b and Porcn expression and canonical Wnt activity marked by Axin2 were induced within 24 h, AT2 proliferation peaked by day 5 as epithelial integrity was restored, and Wnt-related gene expression then returned toward baseline as new AT1 cells appeared (Nabhan et al., 2018). Post-injury Wnt therapy should therefore be guided by contemporaneous cell-state and compartment-specific measurements rather than by a uniform intervention direction. Candidate tools must nevertheless be reversible, locally deliverable, and pharmacodynamically monitorable. CasRx-based RNA regulation and pulmonary lipid nanoparticle (LNP) delivery are complementary: CasRx provides reversible transcript targeting, whereas LNPs can deliver messenger RNA or genome-editing cargo to selected lung cell populations. The combinatorially optimized LNP system reported by Li et al. supported pulmonary mRNA delivery and genome editing in preclinical models (Li et al., 2023). Recent reviews of lung-targeted RNA delivery systems emphasize that inhaled, intravenously targeted, and local pulmonary administration must overcome carrier physicochemical constraints, mucus and surfactant barriers, macrophage uptake, disease-related ventilation heterogeneity, uneven distribution across lung compartments, limited lung tropism, immune responses to repeated dosing, and scalable-manufacturing requirements (Xu et al., 2026).

In the near term, gene editing and RNA regulation are better suited to mechanistic validation than routine treatment. Translation will require pulmonary delivery with controllable exposure, pharmacodynamic readouts such as *AXIN2*, Krt8/CLDN4 transitional-state burden and AT1 maturation markers, selection of regenerative-insufficiency rather than highly fibrotic states, and long-term monitoring for proliferative, immune, bone, and intestinal toxicity.

Among these safety considerations, oncogenic risk warrants particular attention. Persistent dysregulation of canonical Wnt/β-catenin signaling is implicated in tumor initiation and progression (Yu et al., 2021). RSPO3 is a secreted potentiator of canonical Wnt/β-catenin signaling; in a conditional mouse model, sustained *Rspo3* expression amplified Wnt signaling in the intestinal stem-cell niche and induced intestinal adenomas and invasive adenocarcinomas, directly linking chronic Wnt pathway amplification to tumorigenesis (Hilkens et al., 2017). However, because this was a persistent genetic-activation model rather than a drug-exposure study, it does not establish that a finite course of a Wnt-activating drug increases cancer incidence, and current clinical evidence remains insufficient to quantify such a risk. These considerations reinforce the need for short-course modulation and long-term oncologic surveillance.

## 6 Conclusions and perspectives

Wnt signaling acts as a temporal regulator across pulmonary fate specification, branching morphogenesis, postnatal alveologenesis, adult stem-cell niche maintenance, and injury repair. Its defining feature is not a fixed pro-regenerative or pro-fibrotic function, but context-dependent output shaped by developmental timing, anatomical compartment, cell type, and disease stage. Within this framework, BPD can be interpreted as disruption of canonical/noncanonical Wnt switching during the alveologenesis window; COPD often reflects insufficient canonical Wnt-mediated repair in the adult alveolar compartment together with noncanonical Wnt-related repair inhibition and airway remodeling; and IPF represents persistent reactivation of developmental repair programs in abnormal epithelial and fibroblast niches, producing Wnt-TGFβ reinforcing signaling, transitional-state arrest, and matrix deposition. For clinical translation, these issues should be treated as design requirements for precision Wnt modulation rather than as reasons to abandon Wnt-targeted strategies. Future studies should translate experimental time into clinically measurable disease phases because animal models define Wnt dynamics by developmental day or days after injury, whereas patients present with heterogeneous exposure histories, repeated injury, overlapping inflammatory and reparative phases, and variable structural remodeling. Longitudinal sampling, noninvasive imaging, bronchoalveolar lavage analysis, human organoid systems, and spatially resolved tissue studies will therefore be required to assign patients to biologically meaningful Wnt windows and to bridge species differences between synchronized mouse models and chronic, heterogeneous human disease.

Future Wnt-targeted therapies should move beyond global activation or inhibition and instead pursue reversible, local, short-course, and monitorable precision modulation. The central therapeutic question is whether a defined ligand-receptor-cell combination supports epithelial repair, maintains pathological transitional states, activates fibroblasts, or remodels the airway compartment. Frizzled-selective agonists, CBP/β-catenin inhibitors, Wnt5a blockade, PORCN inhibition, CasRx-mediated RNA regulation, and pulmonary lipid nanoparticle delivery provide tool foundations for this approach, but their value will depend on sufficient spatial and temporal control. These requirements favor compartment-selective pulmonary delivery and biomarker-guided patient stratification rather than systemic or long-term pathway manipulation, especially because Wnt/β-catenin signaling participates in oncogenesis and normal tissue homeostasis. Delivery systems should also be evaluated under disease-specific distribution constraints, including ventilation heterogeneity in COPD, matrix stiffening and honeycombing in IPF, and structural immaturity in BPD. Candidate companion biomarker panels should combine indicators such as *FZD4* methylation, *AXIN2* expression, bronchoalveolar lavage Wnt5a/DKK1 or SFRP/DKK ratios, extracellular-vesicle Wnt profiles, Krt8/CLDN4 transitional-state burden, AT1 maturation markers, and spatial nuclear β-catenin rather than relying on any single universal marker. Prospective validation of these biomarkers will be essential before they can guide treatment selection or response monitoring. Integrating single-cell and spatial omics, organoid platforms, pulmonary delivery technologies, and biomarker-stratified clinical trials will be essential for transforming Wnt from a complex developmental pathway into a precision therapeutic target. The practical goal is not to normalize Wnt signaling globally, but to restore the correct Wnt output in the correct cell type, compartment, and temporal window while minimizing oncogenic, fibrotic, and developmental risks.

## References

[B1] Al Wachami N. Guennouni M. Iderdar Y. Boumendil K. Arraji M. Mourajid Y. (2024). Estimating the global prevalence of chronic obstructive pulmonary disease (COPD): a systematic review and meta-analysis. BMC Public Health 24 (1), 297. 10.1186/s12889-024-17686-9 38273271 PMC10811845

[B2] Alapati D. Rong M. Chen S. Hehre D. Hummler S. C. Wu S. (2014). Inhibition of β-catenin signaling improves alveolarization and reduces pulmonary hypertension in experimental bronchopulmonary dysplasia. Am. J. Respir. Cell Mol. Biol. 51 (1), 104–113. 10.1165/rcmb.2013-0346OC 24484510

[B3] Ambalavanan N. Deutsch G. Pryhuber G. Travers C. P. Willis K. A. (2026). The evolving pathophysiology of bronchopulmonary dysplasia. Physiol. Rev. 106 (1), 197–237. 10.1152/physrev.00042.2024 40880213 PMC12483330

[B4] Aros C. J. Pantoja C. J. Gomperts B. N. (2021). Wnt signaling in lung development, regeneration, and disease progression. Commun. Biol. 4 (1), 601. 10.1038/s42003-021-02118-w 34017045 PMC8138018

[B5] Baarsma H. A. Königshoff M. (2017). WNT-er is coming': WNT signalling in chronic lung diseases. Thorax 72 (8), 746–759. 10.1136/thoraxjnl-2016-209753 28416592 PMC5537530

[B6] Baarsma H. A. Skronska-Wasek W. Mutze K. Ciolek F. Wagner D. E. John-Schuster G. (2017). Noncanonical WNT-5A signaling impairs endogenous lung repair in COPD. J. Exp. Med. 214 (1), 143–163. 10.1084/jem.20160675 27979969 PMC5206496

[B7] Barkauskas C. E. Cronce M. J. Rackley C. R. Bowie E. J. Keene D. R. Stripp B. R. (2013). Type 2 alveolar cells are stem cells in adult lung. J. Clin. Invest. 123 (7), 3025–3036. 10.1172/JCI68782 23921127 PMC3696553

[B8] Basil M. C. Alysandratos K. D. Kotton D. N. Morrisey E. E. (2024). Lung repair and regeneration: advanced models and insights into human disease. Cell Stem Cell 31 (4), 439–454. 10.1016/j.stem.2024.02.009 38492572 PMC11070171

[B9] Bottasso-Arias N. Mohanakrishnan M. Trovillion S. Burra K. Russell N. X. Wu Y. (2025). Wnt5a and notum influence the temporal dynamics of cartilaginous mesenchymal condensations in developing trachea. Front. Cell Dev. Biol. 13, 1523833. 10.3389/fcell.2025.1523833 40271154 PMC12015613

[B10] Carlier F. M. Dupasquier S. Ambroise J. Detry B. Lecocq M. Biétry-Claudet C. (2020). Canonical WNT pathway is activated in the airway epithelium in chronic obstructive pulmonary disease. EBioMedicine 61, 103034. 10.1016/j.ebiom.2020.103034 33045470 PMC7559244

[B11] Chen F. Desai T. J. Qian J. Niederreither K. Lü J. Cardoso W. V. (2007). Inhibition of Tgfβ signaling by endogenous retinoic acid is essential for primary lung bud induction. Development 134 (16), 2969–2979. 10.1242/dev.006221 17634193

[B12] Chen F. Cao Y. Qian J. Shao F. Niederreither K. Cardoso W. V. (2010). A retinoic acid-dependent network in the foregut controls formation of the mouse lung primordium. J. Clin. Invest. 120 (6), 2040–2048. 10.1172/JCI40253 20484817 PMC2877937

[B13] Chen X. Zhang C. Wei T. Chen J. Pan T. Li M. (2023). α7nAChR activation in AT2 cells promotes alveolar regeneration through WNT7B signaling in acute lung injury. JCI Insight 8 (15), e162547. 10.1172/jci.insight.162547 37410546 PMC10445688

[B14] Chilosi M. Poletti V. Zamò A. Lestani M. Montagna L. Piccoli P. (2003). Aberrant wnt/Beta-Catenin pathway activation in idiopathic pulmonary fibrosis. Am. J. Pathol. 162 (5), 1495–1502. 10.1016/s0002-9440(10)64282-4 12707032 PMC1851206

[B15] Choi J. Park J. E. Tsagkogeorga G. Yanagita M. Koo B. K. Han N. (2020). Inflammatory signals induce AT2 cell-derived damage-associated transient progenitors that mediate alveolar regeneration. Cell Stem Cell 27 (3), 366–382.e7. 10.1016/j.stem.2020.06.020 32750316 PMC7487779

[B16] Cohen M. L. Brumwell A. N. Ho T. C. Garakani K. Montas G. Leong D. (2024). A fibroblast-dependent TGF-β1/sFRP2 noncanonical wnt signaling axis promotes epithelial metaplasia in idiopathic pulmonary fibrosis. J. Clin. Invest. 134 (18), e174598. 10.1172/jci174598 38980870 PMC11405054

[B17] Conlon T. M. John-Schuster G. Heide D. Pfister D. Lehmann M. Hu Y. (2020). Inhibition of LTβR signalling activates WNT-Induced regeneration in lung. Nature 588 (7836), 151–156. 10.1038/s41586-020-2882-8 33149305 PMC7718297

[B18] Dasgupta C. Sakurai R. Wang Y. Guo P. Ambalavanan N. Torday J. S. (2009). Hyperoxia-induced neonatal rat lung injury involves activation of TGF-β and wnt signaling and is protected by rosiglitazone. Am. J. Physiol. Lung Cell Mol. Physiol. 296 (6), L1031–L1041. 10.1152/ajplung.90392.2008 19304912 PMC3286237

[B19] de Lau W. Peng W. C. Gros P. Clevers H. (2014). The R-spondin/Lgr5/Rnf43 module: regulator of wnt signal strength. Genes Dev. 28 (4), 305–316. 10.1101/gad.235473.113 24532711 PMC3937510

[B20] Desai T. J. Brownfield D. G. Krasnow M. A. (2014). Alveolar progenitor and stem cells in lung development, renewal and cancer. Nature 507 (7491), 190–194. 10.1038/nature12930 24499815 PMC4013278

[B21] Domyan E. T. Ferretti E. Throckmorton K. Mishina Y. Nicolis S. K. Sun X. (2011). Signaling through BMP receptors promotes respiratory identity in the foregut via repression of Sox2. Development 138 (5), 971–981. 10.1242/dev.053694 21303850 PMC4074297

[B22] Duong T. E. Wu Y. Sos B. C. Dong W. Limaye S. Rivier L. H. (2022). A single-cell regulatory map of postnatal lung alveologenesis in humans and mice. Cell Genom 2 (3), 100108. 10.1016/j.xgen.2022.100108 35434692 PMC9012447

[B23] Fang Y. Shao H. Wu Q. Wong N. C. Tsong N. Sime P. J. (2022). Epithelial wntless regulates postnatal alveologenesis. Development 149 (1), dev199505. 10.1242/dev.199505 34931663 PMC8881739

[B24] Frank D. B. Peng T. Zepp J. A. Snitow M. Vincent T. L. Penkala I. J. (2016). Emergence of a wave of wnt signaling that regulates lung alveologenesis by controlling epithelial self-renewal and differentiation. Cell Rep. 17 (9), 2312–2325. 10.1016/j.celrep.2016.11.001 27880906 PMC5214982

[B25] Global Initiative for Chronic Obstructive Lung Disease (2024). Global Strategy for the Diagnosis, Management, and Prevention of Chronic Obstructive Pulmonary Disease: 2024 Report. Bethesda, MD, USA: Global Initiative for Chronic Obstructive Lung Disease (GOLD). Available online at: https://goldcopd.org/2024-gold-report/ (Accessed June 19, 2026).

[B26] Goss A. M. Tian Y. Tsukiyama T. Cohen E. D. Zhou D. Lu M. M. (2009). Wnt2/2b and beta-catenin signaling are necessary and sufficient to specify lung progenitors in the foregut. Dev. Cell 17 (2), 290–298. 10.1016/j.devcel.2009.06.005 19686689 PMC2763331

[B27] Han L. Xu J. Grigg E. Slack M. Chaturvedi P. Jiang R. (2017). Osr1 functions downstream of hedgehog pathway to regulate foregut development. Dev. Biol. 427 (1), 72–83. 10.1016/j.ydbio.2017.05.005 28501478 PMC5519324

[B28] He P. Lim K. Sun D. Pett J. P. Jeng Q. Polanski K. (2022). A human fetal lung cell atlas uncovers proximal-distal gradients of differentiation and key regulators of epithelial fates. Cell 185 (25), 4841–4860.e25. 10.1016/j.cell.2022.11.005 36493756 PMC7618435

[B29] Henderson W. R. Jr Chi E. Y. Ye X. Nguyen C. Tien Y. t. Zhou B. (2010). Inhibition of Wnt/beta-catenin/CREB binding protein (CBP) signaling reverses pulmonary fibrosis. Proc. Natl. Acad. Sci. U. S. A. 107 (32), 14309–14314. 10.1073/pnas.1001520107 20660310 PMC2922550

[B30] Herriges M. Morrisey E. E. (2014). Lung development: orchestrating the generation and regeneration of a complex organ. Development 141 (3), 502–513. 10.1242/dev.098186 24449833 PMC3899811

[B31] Hilkens J. Timmer N. C. Boer M. Ikink G. J. Schewe M. Sacchetti A. (2017). RSPO3 expands intestinal stem cell and niche compartments and drives tumorigenesis. Gut 66 (6), 1095–1105. 10.1136/gutjnl-2016-311606 27511199 PMC5532462

[B32] Hu Y. Ciminieri C. Hu Q. (2021). WNT signalling in lung physiology and pathology. Handb. Exp. Pharmacol. 269, 305–336. 10.1007/164_2021_521 34463851 PMC8969078

[B33] Jin X. Wang J. Cao R. Jiang D. (2020). Wnt signaling pathway: biological function, diseases, and therapeutic interventions. MedComm 7 (1), e70580. 10.1002/mco2.70580 PMC12803509 41541657

[B34] Jirong W. De'an W. Hejing W. Jing L. (2025). Wnt signaling pathway in lung aging and aging-related chronic lung diseases. Biogerontology 27 (1), 23. 10.1007/s10522-025-10367-z 41396493

[B35] Jones D. L. Morley M. P. Li X. Ying Y. Zhao G. Schaefer S. E. (2024). An injury-induced mesenchymal-epithelial cell niche coordinates regenerative responses in the lung. Science 386 (6727), eado5561. 10.1126/science.ado5561 39666855 PMC13159043

[B36] Kadur L. M. P. Sontake V. Tata A. Kobayashi Y. Macadlo L. Okuda K. (2022). Human distal lung maps and lineage hierarchies reveal a bipotent progenitor. Nature 604 (7904), 111–119. 10.1038/s41586-022-04541-3 35355018 PMC9169066

[B37] Khedoe PPSJ van Schadewijk WAAM Schwiening M. Ng-Blichfeldt J. P. Marciniak S. J. Stolk J. (2023). Cigarette smoke restricts the ability of mesenchymal cells to support lung epithelial organoid formation. Front. Cell Dev. Biol. 11, 1165581. 10.3389/fcell.2023.1165581 37795260 PMC10546195

[B38] King T. E. Jr Bradford W. Z. Castro-Bernardini S. Fagan E. A. Glaspole I. Glassberg M. K. (2014). A phase 3 trial of pirfenidone in patients with idiopathic pulmonary fibrosis. N. Engl. J. Med. 370 (22), 2083–2092. 10.1056/nejmoa1402582 24836312

[B39] Kneidinger N. Yildirim A. Ö. Callegari J. Takenaka S. Stein M. M. Dumitrascu R. (2011). Activation of the WNT/β-catenin pathway attenuates experimental emphysema. Am. J. Respir. Crit. Care Med. 183 (6), 723–733. 10.1164/rccm.200910-1560OC 20889911

[B40] Kobayashi Y. Tata A. Konkimalla A. Katsura H. Lee R. F. Ou J. (2020). Persistence of a regeneration-associated, transitional alveolar epithelial cell state in pulmonary fibrosis. Nat. Cell Biol. 22 (8), 934–946. 10.1038/s41556-020-0542-8 32661339 PMC7461628

[B41] Königshoff M. Balsara N. Pfaff E. M. Kramer M. Chrobak I. Seeger W. (2008). Functional wnt signaling is increased in idiopathic pulmonary fibrosis. PLoS One 3 (5), e2142. 10.1371/journal.pone.0002142 18478089 PMC2374879

[B42] Königshoff M. Kramer M. Balsara N. Wilhelm J. Amarie O. V. Jahn A. (2009). WNT1-inducible signaling protein-1 mediates pulmonary fibrosis in mice and is upregulated in humans with idiopathic pulmonary fibrosis. J. Clin. Invest 119 (4), 772–787. 10.1172/JCI33950 19287097 PMC2662540

[B43] Kumawat K. Menzen M. H. Bos I. S. Baarsma H. A. Borger P. Roth M. (2013). Noncanonical WNT-5A signaling regulates TGF-β-induced extracellular matrix production by airway smooth muscle cells. FASEB J. 27 (4), 1631–1643. 10.1096/fj.12-217539 23254341

[B44] Lam A. P. Herazo-Maya J. D. Sennello J. A. Flozak A. S. Russell S. Mutlu G. M. (2014). Wnt coreceptor Lrp5 is a driver of idiopathic pulmonary fibrosis. Am. J. Respir. Crit. Care Med. 190 (2), 185–195. 10.1164/rccm.201401-0079OC 24921217 PMC4226053

[B45] Lecarpentier Y. Gourrier E. Gobert V. Vallée A. (2019). Bronchopulmonary dysplasia: crosstalk between PPARγ, WNT/β-Catenin and TGF-β pathways; the potential therapeutic role of PPARγ agonists. Front. Pediatr. 7, 176. 10.3389/fped.2019.00176 31131268 PMC6509750

[B46] Li C. Xiao J. Hormi K. Borok Z. Minoo P. (2002). Wnt5a participates in distal lung morphogenesis. Dev. Biol. 248 (1), 68–81. 10.1006/dbio.2002.0729 12142021

[B47] Li C. Smith S. M. Peinado N. Gao F. Li W. Lee M. K. (2020). WNT5a-ROR signaling is essential for alveologenesis. Cells 9 (2), 384. 10.3390/cells9020384 32046118 PMC7072327

[B48] Li C. Peinado N. Smith S. M. Zhou J. Gao F. Kohbodi G. (2022). Wnt5a promotes AT1 and represses AT2 lineage-specific gene expression in a cell-context-dependent manner. Stem Cells 40 (7), 691–703. 10.1093/stmcls/sxac031 35429397 PMC9332903

[B49] Li B. Manan R. S. Liang S. Q. Gordon A. Jiang A. Varley A. (2023). Combinatorial design of nanoparticles for pulmonary mRNA delivery and genome editing. Nat. Biotechnol. 41 (10), 1410–1415. 10.1038/s41587-023-01679-x 36997680 PMC10544676

[B50] Lim K. Donovan A. P. A. Tang W. Sun D. He P. Pett J. P. (2023). Organoid modeling of human fetal lung alveolar development reveals mechanisms of cell fate patterning and neonatal respiratory disease. Cell Stem Cell 30 (1), 20–37.e9. 10.1016/j.stem.2022.11.013 36493780 PMC7618456

[B51] Liu J. Pan S. Hsieh M. H. Ng N. Sun F. Wang T. (2013). Targeting Wnt-driven cancer through the inhibition of porcupine by LGK974. Proc. Natl. Acad. Sci. U. S. A. 110 (50), 20224–20229. 10.1073/pnas.1314239110 24277854 PMC3864356

[B52] Liu M. Huo Y. Cheng Y. (2023). Mechanistic regulation of wnt pathway-related progression of chronic obstructive pulmonary disease airway lesions. Int. J. Chron. Obstruct Pulmon Dis. 18, 871–880. 10.2147/copd.s391487 37215745 PMC10198175

[B53] Mammoto T. Chen J. Jiang E. Jiang A. Smith L. E. Ingber D. E. (2012). LRP5 regulates development of lung microvessels and alveoli through the angiopoietin-Tie2 pathway. PLoS One 7 (7), e41596. 10.1371/journal.pone.0041596 22848540 PMC3404972

[B54] Martin-Medina A. Lehmann M. Burgy O. Hermann S. Baarsma H. A. Wagner D. E. (2018). Increased extracellular vesicles mediate WNT5A signaling in idiopathic pulmonary fibrosis. Am. J. Respir. Crit. Care Med. 198 (12), 1527–1538. 10.1164/rccm.201708-1580OC 30044642

[B55] Mayr C. H. Sengupta A. Asgharpour S. Ansari M. Pestoni J. C. Ogar P. (2024). Sfrp1 inhibits lung fibroblast invasion during transition to injury-induced myofibroblasts. Eur. Respir. J. 63 (2), 2301326. 10.1183/13993003.01326-2023 38212077 PMC10850614

[B56] Moreira A. Noronha M. Joy J. Bierwirth N. Tarriela A. Naqvi A. (2024). Rates of bronchopulmonary dysplasia in very low birth weight neonates: a systematic review and meta-analysis. Respir. Res. 25 (1), 219. 10.1186/s12931-024-02850-x 38790002 PMC11127341

[B57] Morrisey E. E. Hogan B. L. (2010). Preparing for the first breath: genetic and cellular mechanisms in lung development. Dev. Cell 18 (1), 8–23. 10.1016/j.devcel.2009.12.010 20152174 PMC3736813

[B58] Mucenski M. L. Wert S. E. Nation J. M. Loudy D. E. Huelsken J. Birchmeier W. (2003). beta-Catenin is required for specification of proximal/distal cell fate during lung morphogenesis. J. Biol. Chem. 278 (41), 40231–40238. 10.1074/jbc.M305892200 12885771

[B59] Nabhan A. N. Brownfield D. G. Harbury P. B. Krasnow M. A. Desai T. J. (2018). Single-cell wnt signaling niches maintain stemness of alveolar type 2 cells. Science 359 (6380), 1118–1123. 10.1126/science.aam6603 29420258 PMC5997265

[B60] Nabhan A. N. Webster J. D. Adams J. J. Blazer L. Everrett C. Eidenschenk C. (2023). Targeted alveolar regeneration with Frizzled-specific agonists. Cell 186 (14), 2995–3012.e15. 10.1016/j.cell.2023.05.022 37321220

[B61] Niehrs C. (2012). The complex world of WNT receptor signalling. Nat. Rev. Mol. Cell Biol. 13 (12), 767–779. 10.1038/nrm3470 23151663

[B62] Nusse R. Clevers H. (2017). Wnt/β-Catenin signaling, disease, and emerging therapeutic modalities. Cell 169 (6), 985–999. 10.1016/j.cell.2017.05.016 28575679

[B63] Okazaki H. Sato S. Koyama K. Morizumi S. Abe S. Azuma M. (2019). The novel inhibitor PRI-724 for Wnt/β-catenin/CBP signaling ameliorates bleomycin-induced pulmonary fibrosis in mice. Exp. Lung Res. 45 (7), 188–199. 10.1080/01902148.2019.1638466 31298961

[B64] Paramore S. V. Trenado-Yuste C. Sharan R. Nelson C. M. Devenport D. (2024). Vangl-dependent mesenchymal thinning shapes the distal lung during murine sacculation. Dev. Cell 59 (10), 1302–1316.e5. 10.1016/j.devcel.2024.03.010 38569553 PMC11111357

[B65] Peng T. Tian Y. Boogerd C. J. Lu M. M. Kadzik R. S. Stewart K. M. (2013). Coordination of heart and lung co-development by a multipotent cardiopulmonary progenitor. Nature 500 (7464), 589–592. 10.1038/nature12358 23873040 PMC3758448

[B66] Qu J. Yue L. Gao J. Yao H. (2019). Perspectives on wnt signal pathway in the pathogenesis and therapeutics of chronic obstructive pulmonary disease. J. Pharmacol. Exp. Ther. 369 (3), 473–480. 10.1124/jpet.118.256222 30952680 PMC6538889

[B67] Raghu G. Remy-Jardin M. Richeldi L. Thomson C. C. Inoue Y. Johkoh T. (2022). Idiopathic pulmonary fibrosis (an update) and progressive pulmonary fibrosis in adults: an official ATS/ERS/JRS/ALAT clinical practice guideline. Am. J. Respir. Crit. Care Med. 205 (9), e18–e47. 10.1164/rccm.202202-0399st 35486072 PMC9851481

[B68] Raslan A. A. Yoon J. K. (2020). WNT signaling in lung repair and regeneration. Mol. Cells 43 (9), 774–783. 10.14348/molcells.2020.0059 32807748 PMC7528681

[B69] Riccetti M. R. Ushakumary M. G. Waltamath M. Green J. Snowball J. Dautel S. E. (2022). Maladaptive functional changes in alveolar fibroblasts due to perinatal hyperoxia impair epithelial differentiation. JCI Insight 7 (5), e152404. 10.1172/jci.insight.152404 35113810 PMC8983125

[B70] Richeldi L. du Bois R. M. Raghu G. Azuma A. Brown K. K. Costabel U. (2014). Efficacy and safety of nintedanib in idiopathic pulmonary fibrosis. N. Engl. J. Med. 370 (22), 2071–2082. 10.1056/nejmoa1402584 24836310

[B71] Ruscitti C. Abinet J. Maréchal P. Meunier M. de Meeûs C. Vanneste D. (2024). Recruited atypical Ly6G^+^ macrophages license alveolar regeneration after lung injury. Sci. Immunol. 9 (98), eado1227. 10.1126/sciimmunol.ado1227 39093958 PMC7616420

[B72] Ruwanpura S. M. Thomas B. J. Bardin P. G. (2020). Pirfenidone: molecular mechanisms and potential clinical applications in lung disease. Am. J. Respir. Cell Mol. Biol. 62 (4), 413–422. 10.1165/rcmb.2019-0328TR 31967851

[B73] Shen S. Wang P. Wu P. Huang P. Chi T. Xu W. (2024). CasRx-based wnt activation promotes alveolar regeneration while ameliorating pulmonary fibrosis in a mouse model of lung injury. Mol. Ther. 32 (11), 3974–3989. 10.1016/j.ymthe.2024.09.008 39245939 PMC11573616

[B74] Shu W. Guttentag S. Wang Z. Andl T. Ballard P. Lu M. M. (2005). Wnt/Beta-Catenin signaling acts upstream of N-myc, BMP4, and FGF signaling to regulate proximal-distal patterning in the lung. Dev. Biol. 283 (1), 226–239. 10.1016/j.ydbio.2005.04.014 15907834

[B75] Skronska-Wasek W. Mutze K. Baarsma H. A. Bracke K. R. Alsafadi H. N. Lehmann M. (2017). Reduced frizzled receptor 4 expression prevents WNT/β-Catenin-driven alveolar lung repair in chronic obstructive pulmonary disease. Am. J. Respir. Crit. Care Med. 196 (2), 172–185. 10.1164/rccm.201605-0904OC 28245136

[B76] Song L. Li K. Chen H. Xie L. (2024). Cell cross-talk in alveolar microenvironment: from lung injury to fibrosis. Am. J. Respir. Cell Mol. Biol. 71 (1), 30–42. 10.1165/rcmb.2023-0426tr 38579159 PMC11225874

[B77] Steimle J. D. Rankin S. A. Slagle C. E. Bekeny J. Rydeen A. B. Chan S. S. K. (2018). Evolutionarily conserved Tbx5-Wnt2/2b pathway orchestrates cardiopulmonary development. Proc. Natl. Acad. Sci. U. S. A. 115 (45), E10615–E10624. 10.1073/pnas.1811624115 30352852 PMC6233116

[B78] Steinhart Z. Angers S. (2018). Wnt signaling in development and tissue homeostasis. Development 145 (11), dev146589. 10.1242/dev.146589 29884654

[B79] Strunz M. Simon L. M. Ansari M. Kathiriya J. J. Angelidis I. Mayr C. H. (2020). Alveolar regeneration through a Krt8^+^ transitional stem cell state that persists in human lung fibrosis. Nat. Commun. 11 (1), 3559. 10.1038/s41467-020-17358-3 32678092 PMC7366678

[B80] Sucre J. M. S. Vickers K. C. Benjamin J. T. Plosa E. J. Jetter C. S. Cutrone A. (2020). Hyperoxia injury in the developing lung is mediated by mesenchymal expression of Wnt5A. Am. J. Respir. Crit. Care Med. 201 (10), 1249–1262. 10.1164/rccm.201908-1513OC 32023086 PMC7233334

[B81] Thébaud B. Goss K. N. Laughon M. Whitsett J. A. Abman S. H. Steinhorn R. H. (2019). Bronchopulmonary dysplasia. Nat. Rev. Dis. Prim. 5 (1), 78. 10.1038/s41572-019-0127-7 31727986 PMC6986462

[B82] Travaglini K. J. Nabhan A. N. Penland L. Sinha R. Gillich A. Sit R. V. (2020). A molecular cell atlas of the human lung from single-cell RNA sequencing. Nature 587 (7835), 619–625. 10.1038/s41586-020-2922-4 33208946 PMC7704697

[B83] Uhl F. E. Vierkotten S. Wagner D. E. Burgstaller G. Costa R. Koch I. (2015). Preclinical validation and imaging of Wnt-induced repair in human 3D lung tissue cultures. Eur. Respir. J. 46 (4), 1150–1166. 10.1183/09031936.00183214 25929950

[B84] Ustiyan V. Bolte C. Zhang Y. Han L. Xu Y. Yutzey K. E. (2018). FOXF1 transcription factor promotes lung morphogenesis by inducing cellular proliferation in fetal lung mesenchyme. Dev. Biol. 443 (1), 50–63. 10.1016/j.ydbio.2018.08.011 30153454 PMC6191344

[B85] Wang Y. Wang L. Ma S. Cheng L. Yu G. (2024). Repair and regeneration of the alveolar epithelium in lung injury. FASEB J. 38 (8), e23612. 10.1096/fj.202400088r 38648494

[B86] Wang Z. Lin J. Liang L. Huang F. Yao X. Peng K. (2025). Global, regional, and national burden of chronic obstructive pulmonary disease and its attributable risk factors from 1990 to 2021: an analysis for the global burden of disease study 2021. Respir. Res. 26 (1), 2. 10.1186/s12931-024-03051-2 39748260 PMC11697803

[B87] Wei X. Qian W. Narasimhan H. Chan T. Liu X. Arish M. (2025). Macrophage peroxisomes guide alveolar regeneration and limit SARS-CoV-2 tissue sequelae. Science 387 (6738), eadq2509. 10.1126/science.adq2509 40048515 PMC12681967

[B88] Wollin L. Wex E. Pautsch A. Schnapp G. Hostettler K. E. Stowasser S. (2015). Mode of action of nintedanib in the treatment of idiopathic pulmonary fibrosis. Eur. Respir. J. 45 (5), 1434–1445. 10.1183/09031936.00174914 25745043 PMC4416110

[B89] Xu S. Li M. Wang T. Chen R. Zhou M. Tang Z. (2026). Lung-targeted RNA delivery systems: strategies and therapeutic applications. J. Nanobiotechnology 24 (1), 142. 10.1186/s12951-025-04019-0 41530739 PMC12888636

[B90] Yates L. L. Schnatwinkel C. Murdoch J. N. Bogani D. Formstone C. J. Townsend S. (2010). The PCP genes Celsr1 and Vangl2 are required for normal lung branching morphogenesis. Hum. Mol. Genet. 19 (11), 2251–2267. 10.1093/hmg/ddq104 20223754 PMC2865378

[B91] Yu F. Yu C. Li F. Zuo Y. Wang Y. Yao L. (2021). Wnt/β-catenin signaling in cancers and targeted therapies. Signal Transduct. Target Ther. 6 (1), 307. 10.1038/s41392-021-00701-5 34456337 PMC8403677

[B92] Zacharias W. J. Frank D. B. Zepp J. A. Morley M. P. Alkhaleel F. A. Kong J. (2018). Regeneration of the lung alveolus by an evolutionarily conserved epithelial progenitor. Nature 555 (7695), 251–255. 10.1038/nature25786 29489752 PMC6020060

[B93] Zepp J. A. Zacharias W. J. Frank D. B. Cavanaugh C. A. Zhou S. Morley M. P. (2017). Distinct mesenchymal lineages and niches promote epithelial self-renewal and myofibrogenesis in the lung. Cell 170 (6), 1134–1148.e10. 10.1016/j.cell.2017.07.034 28886382 PMC5718193

[B94] Zhang J. Liu Y. (2024). Epithelial stem cells and niches in lung alveolar regeneration and diseases. Chin. Med. J. Pulm. Crit. Care Med. 2 (1), 17–26. 10.1016/j.pccm.2023.10.007 38645714 PMC11027191

[B95] Zhang K. Yao E. Lin C. Chou Y. T. Wong J. Li J. (2020). A Mammalian Wnt5a-Ror2-Vangl2 axis controls the cytoskeleton and confers cellular properties required for alveologenesis. eLife 9, e53688. 10.7554/eLife.53688 32394892 PMC7217702

[B96] Zhou Y. Ling T. Shi W. (2024). Current state of signaling pathways associated with the pathogenesis of idiopathic pulmonary fibrosis. Respir. Res. 25 (1), 245. 10.1186/s12931-024-02878-z 38886743 PMC11184855

[B97] Zhou J. Lou X. Wei Z. Mao X. Zhang J. Jin X. (2025). Fzd2 orchestrates canonical and non-canonical wnt signaling to regulate lung development and fibrosis. Cell Commun. Signal 23 (1), 498. 10.1186/s12964-025-02501-8 41257888 PMC12628998

