# Supplementary material for: Temporal regulation of wnt signaling in alveolar development and regeneration: mechanistic insights and therapeutic advances in chronic lung diseases
Source: Front Cell Dev Biol. 2026 Aug 26;14:1914253. doi: 10.3389/fcell.2026.1914253 (PMC13554704; doi:10.3389/fcell.2026.1914253)
Supplement: Supplementary file 1 [file Table1.docx]

Supplementary Table S1. Comparison of injury-induced transitional alveolar epithelial states

| **Dimension** | **DATPs ^[52]^** | **Krt8^+^ ADI ^[53]^** | **PATS ^[54]^** |
| --- | --- | --- | --- |
| Original context | Defined as inflammation-induced, AT2-derived transitional progenitors during mouse alveolar regeneration | Defined as a Krt8^+^ intermediate during mouse alveolar regeneration and compared with related states in human fibrotic lungs | Defined as a transient AT2-to-AT1 intermediate during normal mouse alveolar repair; related PATS-like cells accumulate in human fibrotic lungs |
| Main models/evidence | Mouse bleomycin lineage tracing and AT2 organoids, with DATP-like KRT8^+^/CLDN4^+^ cells observed in human IPF tissue | Time-resolved mouse bleomycin single-cell analysis and lineage tracing, validation in additional mouse injury models, and comparison with human fibrotic tissue | Mouse alveolar organoids, bleomycin injury, AT1-cell ablation, and pneumonectomy with lineage tracing, plus analysis of human fibrotic tissue |
| Possible origin | Predominantly derived from AT2 cells responsive to interleukin-1β in the mouse bleomycin model | AT2 and airway-derived progenitors converge on the Krt8^+^ ADI state in the mouse bleomycin model; airway contribution increases in more severely injured regions | Mouse lineage tracing supports an AT2-to-PATS-to-AT1 trajectory; the origin of PATS-like cells in human fibrosis remains inferred rather than functionally established |
| Representative markers and programs | KRT8 and CLDN4, together with DATP-associated Ndrg1, Sprr1a, and AW112010; p53-, HIF1α/glycolysis-, and inflammatory-response programs; low expression of canonical AT1 and AT2 markers | High KRT8 and KRT18, together with Areg, Hbegf, Itgb6, and Edn1; p53/NF-κB, senescence-associated, and wound-healing programs; low expression of canonical AT1 and AT2 markers | CLDN4, KRT19, and SFN, with CTGF- and LGALS3-associated sub-states; p53-, TGF-β-, DNA-damage-, and transient-senescence programs, with emerging AT1 features |
| Functional implication | Can contribute to AT1 differentiation or return to an AT2 state; sustained interleukin-1β signaling can stall terminal AT1 maturation | A transient regenerative intermediate preceding AT1 differentiation; persistence is associated with fibrotic repair | A lineage-supported AT2-to-AT1 intermediate during normal mouse repair; accumulation of related PATS-like cells is associated with human pulmonary fibrosis |
| Unresolved questions | Whether all DATPs retain regenerative competence or include stressed, non-progenitor subsets | How lineage origin and local injury severity influence ADI fate and reversibility | Whether human PATS-like cells share the lineage, function, and reversibility of mouse PATS |

Abbreviations: ADI, alveolar differentiation intermediate; AT1, alveolar type 1; AT2, alveolar type 2; CTGF, connective tissue growth factor; DATP, damage-associated transient progenitor; HIF, hypoxia-inducible factor; IPF, idiopathic pulmonary fibrosis; NF-κB, nuclear factor-κB; PATS, pre-alveolar type-1 transitional cell state; TGF-β, transforming growth factor-β.
