# Supplementary material for: Temporal regulation of wnt signaling in alveolar development and regeneration: mechanistic insights and therapeutic advances in chronic lung diseases
Source: Front Cell Dev Biol. 2026 Aug 26;14:1914253. doi: 10.3389/fcell.2026.1914253 (PMC13554704; doi:10.3389/fcell.2026.1914253)
Supplement: Supplementary file 2 [file Table2.docx]

Supplementary Table S2. Stage-, compartment-, and mechanism-resolved Wnt disturbances in chronic lung diseases

| **Disease and temporal stage** | **Signal-producing and responding cells/compartments** | **Representative ligands, receptors, and modulators** | **Downstream branch and principal crosstalk** | **Biological and pathological consequences** | **Evidence boundary** |
| --- | --- | --- | --- | --- | --- |
| BPD: late gestation to postnatal alveologenesis | Mesenchymal Wnt5a-associated signals act within the developing AT2–fibroblast–microvascular niche; β-catenin effects vary among epithelial, mesenchymal, and vascular cells | Increased mesenchymal Wnt5a in hyperoxic injury; the decisive cell-specific Wnt5a receptor combination remains unresolved ^[61]^ | Context-dependent canonical β-catenin activity; noncanonical Wnt5a-associated output; crosstalk with PPARγ and TGF-β ^[62-64]^ | Arrested secondary septation, alveolar simplification, impaired microvascular development, and reduced regenerative reserve | Predominantly neonatal rodent/hyperoxia evidence; no validated stage-specific Wnt intervention sequence in preterm infants |
| COPD: adult homeostasis and chronic repair | Alveolar AT2 cells and supporting mesenchyme show regenerative insufficiency, whereas airway epithelium can show a distinct Wnt-active state | Reduced alveolar FZD4; increased mesenchymal Wnt5a/5b with ROR2-related signaling; LTβR constrains Wnt-induced regeneration ^[68-69,71]^ | Reduced alveolar canonical β-catenin responsiveness coexists with noncanonical Wnt5a-associated repair inhibition and canonical activation in airway epithelium ^[68-69,72-73]^ | Failed AT2-mediated repair, emphysema-like tissue loss, basal-cell/mucous remodeling, and small-airway lesions | Human tissue associations are supported by experimental models, but therapeutic receptor activation has not been established in COPD patients |
| IPF: persistent maladaptive repair after adult injury | Abnormal epithelium and Krt8^+^ transitional cells interact with fibroblast foci, Sfrp1^+^ transitional fibroblasts, and Cthrc1^+^ myofibroblasts | LRP5, FZD2, extracellular-vesicle WNT5A, and SFRP1/sFRP2 ^[78-79,81-83]^ | Persistent canonical β-catenin–WISP1/CCN4 output and noncanonical WNT5A/FZD2 signaling reinforce TGF-β-associated fibroblast activation and epithelial metaplasia ^[76,79-82]^ | Transitional-state arrest, invasive myofibroblast differentiation, epithelial metaplasia, fibroblast-foci expansion, and matrix deposition | Strong human-tissue association plus causal preclinical evidence; cell-selective therapeutic windows remain undefined |
